# Antibiotic resistance pattern of *Acinetobacter baumannii* from burns patients: increase in prevalence of *bla*_OXA-24-like_ and *bla*_OXA-58-like_ genes

**Published:** 2019-12

**Authors:** Niloofar Tafreshi, Laleh Babaeekhou, Maryam Ghane

**Affiliations:** Department of Biology, Islamshahr Branch, Islamic Azad University, Islamshahr, Iran

**Keywords:** *Acinetobacter baumannii*, Antibiotic susceptibility, Carbapenem, OXA beta-lactamases

## Abstract

**Background and Objectives:**

Notwithstanding the increased prevalence of *Acinetobacter baumannii* drug-resistant isolates, treatment options are progressively limiting. This study aims to provide a recent report on antibiotic susceptibility in burn wound isolates of *A. baumannii*, and the importance of OXA beta-lactamases in carbapenem resistance.

**Materials and Methods:**

The susceptibility levels to different antimicrobial categories were determined among 84 *A. baumannii* isolates from burn wound infection between 2016 and 2018. Multiplex PCR was used to detect OXA beta-lactamases genes, including *bla*_OXA-51_, *bla*_OXA-23_, *bla*_OXA-24_ and *bla*_OXA-58_. IS*Aba-1* association with *bla*_OXA-51_, *bla*_OXA-23_ and *bla*_OXA-58_ was detected by PCR mapping.

**Results:**

All the isolates were determined as multidrug-resistant (MDR) and 69% as extensively drug-resistant (XDR). Different carbapenems MIC ranges (MIC_50_ and MIC_90_) were observed among the isolates harboring *bla*_OXA-like_ genes and isolates with the OXA-24-like enzyme showed higher carbapenems MIC ranges. The prevalence of *bla*_OXA-51-like_, *bla*_OXA-23-like_, *bla*_OXA-24-like_ and *bla*_OXA-58-like_ were 100%, 53.57%, 41.66% and 30.95%, respectively. IS*Aba-1* insertion sequence was found to be upstream to *bla*_OXA-23-like_ and *bla*_OXA-58-like_ genes in 23 out of 45 (71.1%) *bla*_OXA-23-like_-positive and 4 out of 23 (15.3) *bla*_OXA-58-like_-positive isolates, respectively.

**Conclusion:**

Resistance to carbapenems as the last resort for treatment of *A. baumannii* infections is growing. This study, for the first time in Iran, has observed the increased frequency of *bla*_OXA-24-like_ and *bla*_OXA-58-like_ genes and found an association between IS*Aba-1* and *bla*_OXA-58-like_ gene, which signifies the possible risk of increased diversity in OXA beta-lactamases and growth in carbapenem resistance.

## INTRODUCTION

*Acinetobacter baumannii*, a ubiquitous and opportunistic Gram-negative pathogen, have shown insusceptibility to a wide range of antimicrobial agents, including β-lactamases, which are frequently used in clinical operations (1). Based on the molecular structure, beta-lactamases are subcategorized into four major classes, including A, B, C and D. Classes A to C are both chromosomally encoded and plasmid-encoded enzymes (2). Class D beta-lactamases, known as oxacillinases enzymes or OXA beta-lactamases, are relatively scarce and are identifiable only as plasmid-encoded beta-lactamases. These enzymes have a substrate profile limited to penicillin and oxacillin, though some of them confer resistance to cephalosporins. After considering by Bush et al. (3), the substrate profile of OXA beta-lactamases were designated as 2d, which OXA-1 to OXA-11 are representative enzymes of this class. Carbapenem-hydrolyzing class D beta-lactamases (CHDLs) are a subgroup of class D beta-lactamases that hydrolyze carbapenem antibiotics and have multidrug-resistant to *A. baumannii.*

An increased number of carbapenem-resistant *A. baumannii* isolates harboring *bla*_OXA-23_, *bla*_OXA-24_ (also named *bla*_OXA-40_), and *bla*_OXA-58_ genes have been reported since the 1980s of the last century (3, 4). It is also found that some intrinsic chromosomally encoded OXA-51-like enzymes can mediate resistance to carbapenems when their gene expression is promoted by the environment or mobile genetic elements (5, 6). OXA-23-like enzymes are pinpointed worldwide and are the most widespread OXA-like enzyme in *A. baumannii* (7). In Iran, *bla*_OXA-23_, as the most, and *bla*_OXA-24_, as the less, frequent CHDL-encoding genes are far been reported (8, 9, 10), and evidence has revealed that the *bla*_OXA-58_ gene distribution in Iran and the neighboring countries is less than other CHDL-encoding genes (9–15).

Expression and transformation of the OXA genes could be facilitated by insertion sequences (ISs) such as IS*Aba-1*, IS*Aba-4*, and IS*Aba1-25*, which encode the transposases upstream of *bla*_OXA_ genes and provide an effective promoter for the gene (16). IS*Aba-1*, from IS4 family, has been found to be the upstream of *bla*_OXA-51_, *bla*_OXA-23_ and *bla*_OXA-58_ genes in *Acinetobacter* species (17, 18). It is well documented that resistance to carbapenems mediated by *bla*_OXA-like_ genes can be regulated by the upstream presence of IS*Aba-1* sequence (12, 13, 18). Only a very limited studies in Iran have studied the association of the *bla*_OXA-51_ and *bla*_OXA-23_ genes with IS*Aba-1* (19, 20), and there is no report on the IS*Aba-1* up-regulation of *bla*_OXA-58_ gene.

The emergence of carbapenem resistance mediated by OXA enzymes in *A. baumannii* has demoted the clinical efficacy of this antibiotic, and few studies have unveiled the impact of these enzymes. Hence, the present study was aimed to evaluate the recent antibiotic susceptibility pattern of carbapenem in *A. baumannii* isolates recovered from burn wound infections at a general hospital of Tehran, Iran. This study also verified the presence of *bla*_OXA-23-like_, *bla*_OXA-24-like_ and *bla*_OXA-58-like_ genes and investigated their association with IS*Aba-1*.

## MATERIALS AND METHODS

### Bacterial isolates.

The study included a total of 84 non-repetitive strains of *A. baumannii* isolated from patients with burn wounds in a general hospital of Tehran from 2016 to 2018. The enrolled population was adult patients, of both male and female genders. All patients had serious wound infections that developed to sepsis, uroinfection or pneumonia. The strains were identified by standard microbiological and biochemical techniques (21) and by PCR detection of the intrinsic carbapenemase gene *bla*_OXA-51-like_ (22).

### Ethical issues.

This study was conducted following the approved institutional guidelines of the Islamic Azad Medical University in Tehran (Code: IR.IAU.TMU.REC.1396.279) and volunteer’s data were anonymized before analysis.

### Antibiotic susceptibility test.

Minimal inhibitory concentrations (MICs) for 15 antibiotics were determined by the broth microdilution method according to the Clinical and Laboratory Standards Institute (CLSI) (23) and *Escherichia coli* ATCC 25922 and *Pseudomonas aeruginosa* ATCC 27853 strains were used as references. Initial concentration of antibiotics (AppliChem, Germany) for MIC determination was (256 μg/ml). Antibiotics were selected based on antimicrobial categories proposed by Magiorakos et al. (24) as follows; gentamicin, amikacin, ceftriaxone, cefepime, ciprofloxacin, levofloxacin, ceftazidime, imipenem, meropenem, ploymyxin B, colistin, ampicillin, tetracycline, tigecycline, aztreoam. In this study MDR (multidrug-resistant) was defined as acquired non-susceptibility to at least one agent in three or more antimicrobial categories, XDR (extensively drug-resistant) was defined as non-susceptibility to at least one agent in all but two or fewer antimicrobial categories (i.e. bacterial isolates remain susceptible to only one or two categories) and PDR (pandrug-resistant) was defined as non-susceptibility to all agents in all antimicrobial categories (24).

### *bla*_OXA-like_ genes detection.

Genomic DNA was extracted using the DNA isolation kit (MBST, Iran) as recommended by the manufacturer. Detection of the intrinsic carbapenemase encoding gene *bla*_OXA-51-like_ and three other OXA-carbapenemase genes including *bla*_OXA-23-like_, *bla*_OXA-24-like_ and *bla*_OXA-58-like_ was carried out by multiplex PCR using primers listed in Table 1 (22, 25).

**Table 1.** Sequence of the primers used in this study.

**Target**	**Primer**	**Sequence (5′ to 3′)**	**Amplicon Size (bp)**	**Annealing Temperature (ºC)**	**Reference**
*bla*_OXA-51-like_	OXA-51-F	TAATGCTTTGATCGGCCTTG	353	58	22
	OXA-51-R	TGGATTGCACTTCATCTTGG			
*bla*_OXA-23-like_	OXA-23-F	GATGTGTCATAGTATTCGTCG	1065	58	25
	OXA-23-R	TCACAACAACTAAAAGCACTG			
*bla*_OXA-24-like_	OXA-24-F	GGTTAGTTGGCCCCCTTAAA	246	58	22
	OXA-24-R	AGTTGAGCGAAAAGGGGATT			
*bla*_OXA-58-like_	OXA-58-F	AAGTATTGGGGCTTGTGCTG	599	58	22
	OXA-58-R	CCCCTCTGCGCTCTACATAC			
IS*Aba1- bla*_OXA-51-like_	IS*Aba-1*-F	CACGAATGCAGAAGTTG	1200	60	17, 22
	OXA-51-R	TGGATTGCACTTCATCTTGG			
IS*Aba1- bla*_OXA-23-like_	IS*Aba-1*-F	CACGAATGCAGAAGTTG	1600	60	17, 25
	OXA-23-R	TCACAACAACTAAAAGCACTG			
IS*Aba1- bla*_OXA-58-like_	IS*Aba-1*-F	CACGAATGCAGAAGTTG	1259	60	17, 22
	OXA-58-R	CCCCTCTGCGCTCTACATAC			

The PCR reaction (25 μl) contained 3–5 μl of template DNA, 2.5 μl of 10× PCR buffer, 0.75 μl of 50 mM MgCl_2_, 0.5 μl of 10 mM dNTPs, 0.25 μl of 5 U/μl of Taq DNA polymerase, and 25 pmol of each primers. Primers used and annealing temperatures are given in Table 1. The PCR carried out in a thermocycler (Techne TC512, England) under the following conditions: initial denaturation at 94°C for 5 min followed by denaturation at 94°C for 1 min, annealing at 58°C for 1 min and extension at 72°C for 1 min (30 cycles), and a final extension at 72°C for 7 min. Identified isolates *A. baumannii* (harboring *bla*_OXA-like_ genes), previously reported by Feizabadi et al. (8), were used as positive controls for studied genes. The PCR products were analyzed by electrophoresis on 1.5% agarose gel (Sigma- Aldrich, Germany) containing ethidium bromide (0.5 μg/ml).

### IS*Aba-1* insertion gene detection.

The genetic association between IS*Aba1* sequence and *bla*_OXA-51-like_, *bla*_OXA-23-like_ and *bla*_OXA-58-like_ genes was investigated by PCR mapping using IS*Aba-1* forward (10) and *bla* genes reverse primers (22, 25) (Table 1). The PCR carried out as the *bla*_OXA-like_ multiplex PCR, except that 20 pmol of each primer were used in PCR mixture, and an annealing temperature of 60°C for 45s and extension at 72°C for 3 min was used for 35 cycles of reaction. PCR products were sequenced in the absence of positive controls.

## RESULTS

### Antibiotic susceptibility.

**Fig. 1.**
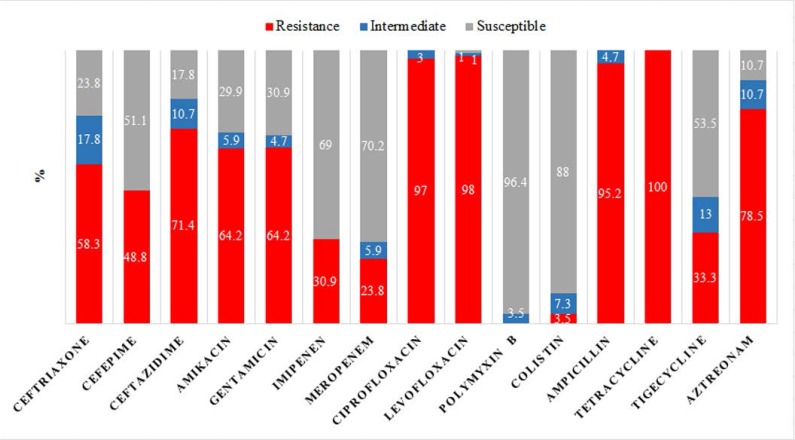
Susceptibility profile in 84 burn wound *A. baumannii* isolates

High percentage of *A. baumannii* isolates were resistant to tetracycline, ciprofloxacin, and levofloxacin, while polymyxin B and colistin were the most effective antibiotics. The results also showed a noticeable resistance rate to cephalosporins and tigecycline. The total antibiotic resistance results are presented in Fig. 1. According to the epidemiological definition, all 84 isolates (100%) were grouped as MDR, whereas 58 isolates (69%) were considered as XDR and none of the isolates were grouped as PDR. Different carbapenems MIC ranges (MIC_50_ and MIC_90_) were observed in the isolates harboring *bla*_OXA-like_ genes. Among them, isolates with the OXA-24-like enzyme showed higher carbapenems MIC ranges (Table 2).

**Table 2.** MIC values (μg/ml) for imipenem and meropenem in studied *A. baumannii* isolates harboring *bla*_OXA-like_ genes.

**Gene**	**Number**	**MIC_50_**		**MIC_90_**		**MIC range**	

		**IM**	**MEM**	**IM**	**MEM**	**IM**	**MEM**
*bla*_OXA-23_	45	0.5	0.5	16	8	0.062–32	0.032–32
IS*Aba-1* –upstream *bla*_OXA-23_	32 out of 45	4	2	16	8	0.062–32	0.032–32
*bla*_OXA-24_	35	1	1	32	16	0.032–32	0.032–16
*bla*_OXA-58_	26	1	0.5	16	16	0.062–32	0.032–32
*bla*_OXA-23_ only	23	0.5	0.25	2	4	0.062–16	0.032–8
*bla*_OXA-24_ only	23	0.5	0.5	32	16	0.032–16	0.032–16

Notes: Breakpoints of IM and MEM, MIC ≤2 μg/mL: sensitive; MIC =4 μg/mL: intermediate; MIC ≥8 μg/mL: resistant (CLSI, 2017).

IM: Imipenem, MEM: Meropenem

### Distribution of *bla*_OXA-like_ genes.

**Fig. 2.**
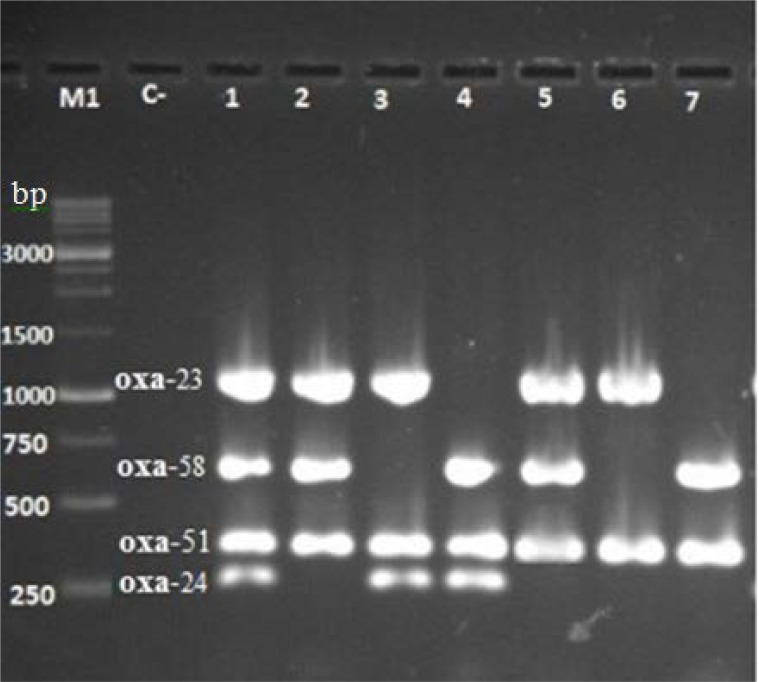
Detection of *bla*_OXA-like_ genes from *A. baumannii* isolates by multiplex PCR amplification. Lanes 1 to 7: isolates harboring *bla*_OXA-like_ genes. Lane C-: no chromosomal DNA (negative control). Lane M1: 1-kb DNA ladder (SINACLON, Iran).

Multiple PCR reactions by *bla*_OXA-like_ genes specific primers produced DNA fragments of 353, 1065, 246 and 599 bp, from *bla*_OXA-51-like_, *bla*_OXA-23-like_, *bla*_OXA-24-like_ and *bla*_OXA-58-like_ genes respectively (Fig. 2). The prevalence of *bla*_OXA-51-like_, *bla*_OXA-23-like_, *bla*_OXA-24-like_ and *bla*_OXA-58-like_ were 100%, 53.57%, 41.67% and 30.95%, respectively. The co-existence of *bla*_OXA-51_, *bla*_OXA-23_ and *bla*_OXA-24_ was observed in 3.57% (3/84) of isolates.

Co-existence of *bla*_OXA-51_, *bla*_OXA-23_, *bla*_OXA-58_ and *bla*_OXA-51_, *bla*_OXA-24_, *bla*_OXA-58_ was seen in 20.23% (17/84) and 10.7% (9/84) of isolates respectively. There was an isolate (1.19%) that carried all four genes. Six isolates from 84 (7.14%) carried only the *bla*_OXA-51_ gene. The distribution of *bla*_OXA_-types among carbapenem-resistant *A. baumannii* isolates is shown in Table 3.

**Table 3.** *bla*_OXA-like_ genes and IS*Aba-1* insertion sequence distribution among imipenem and/or meropenem resistant *A. baumannii* isolates (n=28).

Isolates^*^	***bla*_OXA-like_ gene**				

	**51**	**23**	**24**	**58**	**IS*Aba-1* upstream**
1	+	−	+	+	
3	+	−	+	−	
4	+	+	−	+	*bla*_OXA-23_
5	+	+	−	−	*bla*_OXA-23_
6	+	−	+	−	
10	+	+	−	−	*bla*_OXA-23_
14	+	+	−	+	*bla*_OXA-23_
21	+	−	+	−	
25	+	+	−	−	*bla*_OXA-23_
32	+	−	+	−	
33	+	−	−	−	*bla*_OXA-51_
50	+	+	−	+	*bla*_OXA-23_
53	+	+	−	−	*bla*_OXA-23_
59	+	+	−	−	*bla*_OXA-23_
65	+	−	+	+	
73	+	−	+	−	
74	+	−	+	−	
75	+	+	−	+	*bla*_OXA-23_
76	+	+	−	−	*bla*_OXA-23_
78	+	+	−	+	*bla*_OXA-23_
79	+	−	−	−	
85	+	−	+	−	
90	+	−	+	−	
91	+	−	+	−	
92	+	+	+	−	*bla*_OXA-23_
93	+	+	−	−	*bla*_OXA-23_
97	+	−	+	−	
100	+	−	−	+	

* Isolate numbering is not under the total number of studied isolates

### IS*Aba-1* element association.

**Fig. 3.**
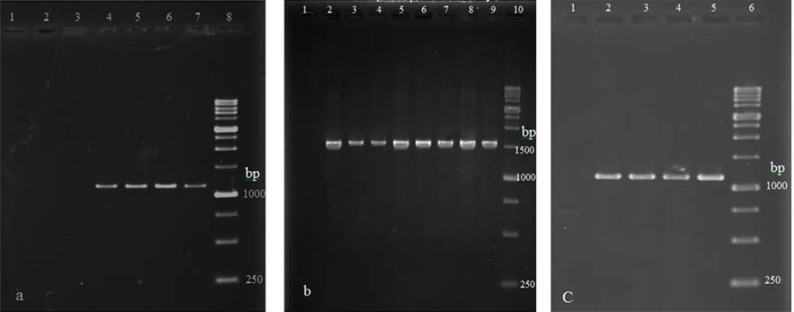
PCR products obtained using the IS*Aba-1* forward primer (IS*Aba-1*F) and *bla*_OXA-like_ gene reverse primers. (a) Carbapenem-resistant isolates carrying only *bla*_OXA-51-like_ after amplification with the IS*Aba-1*F and OXA-51-R primer pair. Lane 1: no chromosomal DNA (negative control). Lanes 2 and 3: DNA of isolates failed to give a band. Lanes 4–7: DNA of isolates with IS*Aba-1* upstream of *bla*_OXA-51-like_ gene with the 1200-bp PCR product. Lane 8: 1-kb DNA ladder. (b) Lane 1: no chromosomal DNA (negative control). Lanes 2–9: DNA of isolates with IS*Aba-1* upstream *bla*_OXA-23-like_ gene with the 1600-bp PCR product. Lane 10: 1-kb DNA ladder. (c) Lane 1: no chromosomal DNA (negative control). Lanes 2–5: DNA of isolates with IS*Aba-1* upstream of *bla*_OXA-58-like_ gene with the 1259-bp PCR product. Lane 6: 1-kb DNA ladder.

Among carbapenem-resistant isolates with only *bla*_OXA-51-like_, 4 isolates yielded a band of 1200 bp in a PCR reaction using IS*Aba-1* forward primer and the *bla*_OXA-51-like_ reverse primer (Fig. 3). Isolates with positive PCR products for *bla*_OXA-23-like_ and *bla*_OXA-58-like_ genes showed a band of 1600 and 1259 bp respectively in PCR reactions using forward primer for IS*Aba-1* and the reverse primers for *bla*_OXA-51-like_ and *bla*_OXA-58-like_ genes (Fig. 3). The IS*Aba-1* element was found upstream *bla*_OXA-23-like_ and *bla*_OXA-58-like_ genes in 32 out of 45 (71.11%) and 4 out of 26 (15.38%) *A. baumannii* isolates respectively. IS*Aba-1* insertion sequence presence among 28 carbapenem-resistant is shown in Table 3.

## DISCUSSION

The present study showed a remarkable resistance of *A. baumannii* isolates to the tested antibiotics. The significance of these results becomes greater when using third and fourth-generation antibiotics, such as cephalosporins and carbapenems, for the treatment of *A. baumannii* infections. Almost all the isolates studied here indicated susceptibility to polymyxin B and colistin and similar result to that obtained by several numbers of previous studies conducted in Iran (26, 9, 27, 28). Our study found the susceptibility of 56 out of 58 XDR *A. baumannii* isolates to polymyxin B, meaning that polymyxin B has ability to re-emerge in medical practice for the treatment of infections caused by MDR and XDR *A. baumannii* strains. In a clinical study, it has been suggested that polymyxin B alone or in combination with other antibiotics can decrease the overall mortality and also can remove bacteria from patients. However, further investigations on the pharmacokinetics, pharmacodynamics, and toxicodynamics of polymyxin B are needed to find the appropriate doses of the drug (29). Aztreonam, a superior antibiotic to ceftazidime and more stable than carbapenemases (30), was tested in this study and indicated a high prevalence of resistance. The result is comparable with those of other studies (9, 26) and suggest to eliminate aztreonam from the list of therapeutic solutions for *A. baumannii* infections control.

The carbapenem-resistant isolates in the present work could be attributed to the frequent use of carbapenems, especially after explosive dissemination of ESBLs and CTX-M pandemic. Besides, this behavior may arise from multiple mechanisms of carbapenem resistance in *A. baumannii* and dissemination of carbapenemases by the acquisition of plasmid/chromosome-mediated resistance genes (4). It is noteworthy to say that, all imipenem- and meropenem-resistant *A. baumannii* strains of this study were identified as MDR and XDR.

To assay OXA beta-lactamases as a carbapenem resistance mechanism, the isolates were screened for the most prevalent *bla*_OXA_ genes, including *bla*_OXA-23-like_, *bla*_OXA-24-like_ and *bla*_OXA-58-like_ (4). The products of these genes are consistently associated with resistance or at least reduced susceptibility to carbapenems (4). The isolates were also screened for *bla*_OXA-51-like_, a prevalent and an intrinsic gene in *A. baumannii* species with the chromosomal origin that has a relatively weak ability to hydrolyze carbapenems (4, 5). *bla*_OXA-like_ genes are candidates for IS*Aba-1* acquisition, which is commonly associated with the expression of CHDL-encoding genes in *A. baumannii* and can contribute to carbapenemase genes spread among *Acinetobacter* species (4, 16). To survey this, the association of IS*Aba-1* with *bla*_OXA-51-like_ (in *bla*_OXA-23-like_-, *bla*_OXA-24-like_- and *bla*_OXA-58-like_-negative isolates), *bla*_OXA-23-like_ and *bla*_OXA-58-like_ genes were also investigated in our isolates.

The *bla*_OXA-23-like_ gene distribution frequency among all the studied *A. baumannii* isolates was the most, which coordinates with other studies in different regions of Iran and some neighboring countries (9–15, 19, 20, 28, 31). There were increased MIC values for imipenem and meropenem in *A. baumannii* isolates harboring *bla*_OXA-23_ gene, especially in ones with upstream IS*Aba-1* element (Table 2). Meanwhile, from 13 carbapenem-resistant *A. baumannii* harboring *bla*_OXA-23-like_ all but one showed upstream IS*Aba-1* element, (Table 3). These observations emphasize the *bla*_OXA-23-like_ gene role and its up-regulation by IS*A-ba-1* element as a major mechanism for carbapenem resistance phenotype.

OXA-24-like carbapenemase is widely disseminated, but its prevalence is less than OXA-23-like (16). OXA-24-like frequency among *A. baumannii* isolates has formerly been reported in Iran and some neighboring countries. Having looked at previous studies, we found that over time, *bla*_OXA-24-like_ gene distribution has increased (9–15, 19, 20, 28, 31). In the present study, the spread of the *bla*_OXA-24-like_ gene (41.66%) was noticeably higher than the rates reported previously (9–15, 19, 20, 28, 31). A possible explanation for such elevation could be the cephalosporin antibiotics overuse, especially because almost all the *A. baumannii* isolates harboring *bla*_OXA-24-like_ genes were resistant to ceftriaxone, cefepime, and ciprofloxacin. According to the results summarized in Table 2, *A. baumannii* isolates harboring the *bla*_OXA-24-like_ gene showed higher MIC values than those with *bla*_OXA-23-like_. This evidence reflects the higher contribution of the *bla*_OXA-24-like_ gene in carbapenem resistance than *bla*_OXA-23-like_, which is in accordance with a recent study (32).

*bla*_OXA-58-like_ gene was identified in 30.95% of the studied isolates, which was remarkably higher than other reports in Iran (9–11, 20, 28) and neighboring countries (12–15, 31). Association of IS*Aba-1* gene with the *bla*_OXA-58-like_ in *A. baumannii* isolates of the present study, which was observed for the first time in Iran, is a justification for its growth in dissemination and suggests the possibility of wider dissemination of *bla*_OXA-58-like_ gene.

In this study, from 28 carbapenem-resistant *A. baumannii* isolates, 27 harbored *bla*_OXA-23-like_ and/or *bla*_OXA-24-like_ and/or *bla*_OXA-58-like_ genes which signifies the responsibility of these genes in carbapenem resistance. However, examining the higher number of carbapenem-resistant *A. baumannii* isolates could assist to come to a stronger conclusion regarding the importance of OXA β-lactamases in carbapenem resistance.

In conclusion, the high frequency of MDR and XDR *A. baumannii* strains, in the present study, represents the wide dissemination of antibiotic-resistant *A. baumannii* strains in the healthcare centers of Iran and only polymyxin B, colistin, imipenem, and meropenem can be considered as effective drugs for the treatment of *A. baumannii* infection. This problem can be managed by Iran’s annual reports on drug resistance to the World Health Organization (WHO) and Central Asian and Eastern European Surveillance of Antimicrobial Resistance (CAESAR). Our results provided evidence for higher prevalence of *bla*_OXA-24-like_ and *bla*_OXA-58-like_ genes than past and the association of the *bla*_OXA-58-like_ gene with the IS*Aba-1* insertion sequence can speculate a shift in *bla*_OXA-like_ genes distribution in Iran. More studies are required to strengthen the proposed hypothesis. As carbapenems are not as toxic as colistin, it is essential to preserve their efficacy for clinical success against *A. baumannii*. This goal may achieve by accurate monitoring of resistance mechanisms to carbapenems.
